# The Ghrelin/GHSR-1a Axis Attenuates Preeclampsia-like Features with Decidual Macrophage Reprogramming and Improved Placental Remodeling

**DOI:** 10.3390/biom16060809

**Published:** 2026-05-29

**Authors:** Lingling Zhang, Jiani Yuan, Ningning Hu, Jian Yu, Liwen Zhang, Rujun Chen, Xiaoqin Wang

**Affiliations:** 1 Department of Obstetrics and Gynecology, Shanghai Fifth People’s Hospital, Fudan University, Shanghai 200240, China; 23211290017@m.fudan.edu.cn (L.Z.); yuanjiani@fudan.edu.cn (J.Y.); hax199006@gmail.com (N.H.); 13321837079@163.com (L.Z.); 2 Center of Community-Based Health Research, Fudan University, Shanghai 200240, China; 3 Department of Central Laboratory, Shanghai Fifth People’s Hospital, Fudan University, Shanghai 200240, China; possimonize@gmail.com

**Keywords:** ghrelin/GHSR-1a axis, macrophage polarization, maternal–fetal interface, Preeclampsia, placenta

## Abstract

Preeclampsia (PE) is a severe pregnancy-specific hypertensive disorder characterized by immune microenvironment dysregulation at the maternal–fetal interface, with decidual macrophage phenotypic imbalance being a key pathological feature. The Ghrelin/growth hormone secretagogue receptor-1a (GHSR-1a) axis exerts immunomodulatory and anti-inflammatory effects, but its role in regulating decidual macrophage infiltration and phenotypic marker expression in PE remains unclear. In this study, we first detected the expression of the Ghrelin/GHSR-1a axis in decidual tissues from 10 healthy pregnant women and 12 PE patients via immunohistochemistry (IHC). We then established a lipopolysaccharide (LPS)-induced PE-like rat model to investigate the axis’s functional role and underlying mechanisms. Intriguingly, clinical analysis revealed a severity-dependent compensatory escalation of the Ghrelin/GHSR-1a axis in PE decidual tissues, potentially representing an endogenous antagonistic response to pregnancy-associated pathological stress. In the animal model, exogenous Ghrelin supplementation reversed LPS-induced PE-like phenotypes, including hypertension, proteinuria, fetal growth restriction (FGR), and placental dysfunction, and alleviated pathological damage to the maternal liver, kidney, and placenta. Mechanistically, Ghrelin modulated decidual macrophage phenotypic marker expression by downregulating the M1 marker CD86 and upregulating the M2 marker CD163 and promoted trophoblast invasion and spiral artery remodeling by restoring laminin, α-cytokeratin 7 (α-CK7), and α-smooth muscle actin (α-SMA) expression in placental tissue. All protective effects of Ghrelin were abrogated by co-administration of D-lys-3-GHRP-6, a specific GHSR-1a antagonist, confirming the dependence on the Ghrelin/GHSR-1a axis. Collectively, our findings suggest that the Ghrelin/GHSR-1a axis is compensatorily upregulated in PE and may exert a protective role by regulating decidual macrophage phenotypic marker expression and improving placental function, providing preliminary evidence that this axis merits further investigation as a potential research target for PE.

## 1. Introduction

Preeclampsia (PE) is a severe pregnancy-specific hypertensive disorder that affects 2–8% of all pregnancies worldwide, with its annual incidence on the rise [1,2]. Clinically, PE is defined as gestational hypertension emerging after 20 weeks of gestation, accompanied by proteinuria or multiple organ dysfunction [2,3]. As the second leading cause of global direct maternal mortality, PE is closely associated with adverse perinatal outcomes, including fetal growth restriction (FGR), preterm birth, and even maternal–fetal death in severe cases [4]. To date, delivery remains the only definitive treatment for PE, and effective clinical interventions remain limited for high-risk populations [4,5]. Therefore, there is an urgent need to clarify the pathophysiological mechanisms of PE, among which the maternal–fetal interface and local immune microenvironment have become core research focuses [6].

The pathogenesis of PE is complex and multifactorial, with the two-stage hypothesis being widely accepted as a core framework for understanding its development [7,8]. The first stage is characterized by defective trophoblast invasion and insufficient spiral artery remodeling in early pregnancy, resulting in placental ischemia and hypoxia. The second stage involves the release of placental-derived factors such as soluble fms-like tyrosine kinase-1 (sFlt-1) into maternal circulation, triggering systemic endothelial injury, inflammation, and oxidative stress [9]. Key molecules involved in this cascade include sFlt-1, which binds to placental growth factor (PlGF) and vascular endothelial growth factor (VEGF) to block their pro-angiogenic effects, resulting in impaired vascular relaxation and increased blood pressure [10,11]; nitric oxide (NO) signaling, whose dysregulation plays a pivotal role in promoting vasoconstriction and driving the development of hypertensive phenotypes [12,13]; and heme oxygenase/hydrogen sulfide pathways, which exert protective roles in maintaining vascular homeostasis and suppressing inflammation and are downregulated in PE [14]. Notably, systemic inflammation and immune dysregulation are central to the “second stage” of PE pathogenesis.

Macrophages are the major immune cell population at the maternal–fetal interface, participating in spiral artery remodeling, trophoblast invasion regulation, and immune homeostasis maintenance during early pregnancy [15]. Macrophages are polarized into pro-inflammatory M1 and anti-inflammatory M2 subtypes with high phenotypic plasticity. Normal pregnancy is dominated by M2 macrophage polarization to maintain maternal–fetal immune tolerance and normal placentation [16]. In contrast, PE is characterized by a severe M1/M2 polarization imbalance at the maternal–fetal interface, with upregulated expression of M1-specific markers and downregulated M2 markers [16,17]. Such imbalance suppresses trophoblast invasion, impairs spiral artery remodeling and exacerbates local inflammation, leading to placental hypoperfusion and PE onset. Nevertheless, the key regulatory molecules and molecular mechanisms governing decidual macrophage polarization in PE remain poorly understood.

Ghrelin is a 28-amino-acid polypeptide hormone that exerts its biological functions primarily by binding to its functional receptor, growth hormone secretagogue receptor-1a (GHSR-1a) [18]. Beyond regulating appetite and energy metabolism, Ghrelin is abundantly expressed in placental trophoblasts with gestational dynamic expression, implying its involvement in pregnancy maintenance and fetal development [19]. Emerging evidence confirms that Ghrelin possesses potent immunomodulatory capacity, regulating macrophage polarization toward the M2 phenotype and alleviating pathological inflammation in multiple diseases [20,21]. However, whether Ghrelin modulates decidual macrophage polarization at the maternal–fetal interface via the GHSR-1a pathway, and thereby participates in the pathogenesis and progression of PE, remains unelucidated.

In this study, we detected the clinical expression pattern of the Ghrelin/GHSR-1a axis in decidual tissues from healthy pregnant women and PE patients. Using an LPS-induced PE-like rat model, we further explored the effects of this axis on PE-like phenotypes, decidual macrophage phenotypic profile, trophoblast invasion and spiral artery remodeling. We also verified the protective role of recombinant Ghrelin and the antagonistic effect of D-lys-3-GHRP-6. This study aims to elucidate the role of the Ghrelin/GHSR-1a axis in PE pathogenesis and provide novel potential therapeutic targets for PE.

## 2. Materials and Methods

### 2.1. Study Design and Participants

A prospective observational case–control study was conducted at the Shanghai Fifth People’s Hospital Affiliated to Fudan University from June 2025 to December 2025. Consecutive inpatients with singleton pregnancy undergoing cesarean section were enrolled in this study, with no convenience sampling applied; all eligible patients meeting the predefined inclusion and exclusion criteria were included in the study population. A total of 22 singleton pregnant women who underwent cesarean section were enrolled, including 10 healthy pregnant women (control group) and 12 PE patients, including 7 cases of mild PE and 5 cases of severe PE. Given the low annual incidence of severe PE (≈5%) in the cesarean section population of our hospital and the constraints of clinical resource collection, a consecutive sampling strategy was adopted for subject enrollment. Post hoc power analysis was performed using G*Power 3.1 software, which confirmed that the final sample size (n = 22) could provide 80% statistical power to detect a medium effect size (Cohen’s d = 0.8) for key clinical outcome indicators (e.g., gestational age at delivery, baseline BMI) at a two-sided α = 0.05. This study was designed as a pilot investigation to explore the preliminary clinical correlation between the Ghrelin/GHSR-1a axis and PE severity.

PE was diagnosed according to the latest clinical criteria [1]: hypertension (SBP ≥ 140 mmHg and/or DBP ≥ 90 mmHg) onset after 20 weeks of gestation, accompanied by random urine protein ≥++, a urine protein/creatinine ratio ≥ 0.3, or 24 h urine protein ≥ 0.3 g. Severe PE was defined as PE complicated with any of the following: SBP ≥ 160 mmHg and/or DBP ≥ 110 mmHg; thrombocytopenia (<100 × 10^9^/L); liver dysfunction (serum transaminase levels ≥ 2 times the upper limit of normal); severe persistent right upper quadrant/epigastric pain without other identifiable causes; renal impairment (serum creatinine > 1.1 mg/dL or ≥2 times the baseline level without other renal diseases); pulmonary edema; new-onset headache unrelieved by analgesics; or visual disturbances.

Inclusion criteria: (1) age 20–40 years; (2) singleton pregnancy; (3) cesarean delivery; (4) complete clinical and follow-up data. Exclusion criteria: (1) chronic essential hypertension, autoimmune diseases, or other underlying medical/surgical conditions; (2) gestational diabetes mellitus (GDM); (3) placental abruption; (4) smoking history; (5) multiple pregnancy.

This study was approved by the Medical Ethics Committee of the Shanghai Fifth People’s Hospital, Fudan University (No. 2024242, 18 December 2024) and conducted in accordance with the Declaration of Helsinki. Informed written consent was obtained from all participants prior to enrollment. A flowchart of participant screening and enrollment was generated using WPS Office (Version 12.1, Kingsoft Office Corporation, Zhuhai, China) and is provided in the Supplementary Material (Figure S1), which detailed the number of screened subjects, exclusion reasons and corresponding case numbers, and the final grouping of enrolled subjects.

### 2.2. Collection of Decidual Tissue

Decidual tissue samples were collected by a single experienced obstetrician to ensure standardization of the sampling operation across all cases. Sampling was performed within 5 min after fetal delivery and before placental separation during cesarean section, under sterile surgical conditions to avoid maternal blood and placental villous contamination. The decidua basalis at the upper segment of the uterine body, the main implantation site of the placenta with the most abundant decidual stromal cells and immune cells, was selected as the unified sampling site for all participants. Sampling was limited to the decidual tissue layer with a sampling volume of 0.5 cm × 0.5 cm × 0.3 cm for each case to ensure consistency. The purified decidual tissue was immediately fixed in 4% paraformaldehyde (4 °C, pH 7.4) within 10 min after sampling, with a fixative volume 10 times the tissue volume, and fixed for 24 h at 4 °C. After fixation, the tissue was subjected to routine gradient dehydration, xylene transparency, and paraffin embedding. Serial 5 μm thick coronal sections were cut using a microtome (Leica, Wetzlar, Germany) and mounted on poly-L-lysine-coated glass slides for subsequent immunohistochemical (IHC) staining and analysis. A small portion of each decidual tissue sample was randomly selected for frozen section staining to verify the purity of the decidual tissue before paraffin embedding; samples with contamination were discarded and re-sampled immediately.

### 2.3. Immunohistochemical (IHC) Staining

Paraffin sections were subjected to routine dewaxing in xylene and rehydration in a graded ethanol series. Antigen retrieval was performed by boiling the sections in EDTA buffer (pH 9.0) for 15 min, followed by natural cooling to room temperature. Endogenous peroxidase activity was blocked with 3% hydrogen peroxide at room temperature for 20 min, and non-specific protein binding was blocked with 10% goat serum (in PBS) at room temperature for 20 min. The sections were then incubated with primary antibodies against Ghrelin (04010006823, 1:200, Servicebio, Wuhan, China), GHSR-1a (860429, 1:200, Servicebio, Wuhan, China), and Laminin (AY2822S, 1:200, Servicebio, Wuhan, China) at 4 °C overnight in a humidified chamber. After washing three times with PBS (5 min each), the sections were incubated with horseradish peroxidase (HRP)-conjugated secondary antibodies (1:500) at room temperature for 45 min. Diaminobenzidine (DAB) chromogenic solution (100 μL per section) was added for color development, and the reaction was terminated with tap water under microscopic observation. The sections were counterstained with hematoxylin for 30 s, differentiated with 1% hydrochloric acid–ethanol, blued with 0.5% ammonia water, and then dehydrated, cleared, and mounted with neutral balsam. PBS was used instead of primary antibodies as the negative control, and isotype-matched IgG was used as an additional specific negative control; human gastric mucosa tissue with high Ghrelin/GHSR-1a expression was used as the positive control. All stained sections were photographed under a light microscope (Olympus, Tokyo, Japan) at fixed magnification, exposure time, and light intensity for subsequent quantitative analysis. The integrated optical density (IOD) of positive staining was quantified using ImageJ software (Version 1.8.0, National Institutes of Health, Bethesda, MD, USA), with a fixed threshold based on the negative control and automatic background correction by the software. Next, 4–6 random visual fields per section were selected for IOD measurement, and the average value was taken as the final IOD of the sample. All quantitative analyses were performed by two independent experimenters blinded to the clinical grouping, and disagreements were resolved by joint review. Internal reference was used for inter-slide normalization to ensure the consistency of staining results across different slides.

### 2.4. Establishment and Grouping of Animal Models

A total of 24 healthy pregnant Sprague Dawley rats with a body weight of 220–250 g and gestational day 0 (GD0) confirmed by vaginal plug detection were purchased from the Experimental Animal Center of Fudan University (Shanghai, China) and randomly divided into four groups (n = 6 per group) using a random number table method; the cages were randomly placed in the animal facility to avoid environmental bias. All intervention administrations and subsequent indicator detections were performed by experimenters blinded to the group allocation. All rats were subjected to adaptive operation training with gentle grasping and fixation for 3 days before drug administration to reduce stress caused by injection operations: (1) Control group: Tail vein injection of normal saline (1 mL/kg) once daily at 06:00 from GD5 to GD20, matching the injection route and frequency of LPS; (2) LPS group: Tail vein injection of LPS (1.0 μg/kg, *Escherichia coli* O55:B5, Sigma-Aldrich, St. Louis, MO, USA) once daily at 06:00 from GD5 to GD20 to establish the PE-like model; (3) LPS+Ghrelin group: Tail vein injection of LPS (1.0 μg/kg) at 06:00, plus intraperitoneal injection of Ghrelin (100 μg/kg, Sigma-Aldrich, St. Louis, MO, USA) twice daily (08:00 and 20:00) from GD5 to GD20; (4) LPS+Ghrelin+D-lys-3-GHRP-6 group: Tail vein injection of LPS (1.0 μg/kg) at 06:00, plus intraperitoneal injection of Ghrelin (100 μg/kg) and subcutaneous injection of D-lys-3-GHRP-6 (BH-10461, 6 mmol/day, Sigma-Aldrich, Sigma-Aldrich, St. Louis, MO, USA, a specific GHSR-1a antagonist) twice daily (08:00 and 20:00) from GD5 to GD20.

All rats were housed in a specific pathogen-free (SPF) animal facility under standardized conditions (temperature: 25 ± 1 °C, humidity: 50% ± 5%, 12 h light/dark cycle) with free access to standard chow and sterile water. The PE-like model was considered successful if the rat’s systolic blood pressure (SBP) increased by >30 mmHg compared with the control group and SBP ≥ 115 mmHg (compared with the baseline SBP of normal pregnant SD rats (90–100 mmHg)). The animal study protocol was approved by the Chedun Laboratory Animal Ethics Committee (No. AD20240843, 30 August 2024).

### 2.5. Measurement of Blood Pressure

Systolic blood pressure (SBP) and diastolic blood pressure (DBP) of all pregnant rats were measured every 2–3 days from GD0 to GD20 between 08:00 and 12:00 under quiet conditions. All rats received adaptive training for 3 days before baseline measurement. Prior to measurement, rats were fixed in a rat restraint bag and placed in a constant temperature incubator (37–39 °C) for 10 min to reduce stress. The rat tail was passed through a non-invasive tail-cuff blood pressure sensor (BP-98A, Softron, Beijing, China), with the sensor fixed at the root of the tail to ensure tight contact with the caudal artery. After the blood pressure waveform stabilized (fluctuation < 10 mmHg), low-quality cycles with severe fluctuations were discarded; a total of 5–6 measurement cycles were initially collected, and 3 stable cycles were selected for averaging as the final result. All measurements were performed by the same experimenter who was blinded to the group allocation.

### 2.6. Determination of Urinary Protein and Urine Output

From GD0 to GD20, rats were placed in metabolic cages (with fecal–urinary separation filters) every 2–3 days from 20:00 to 10:00 the next day (14 h fasting period). Rats had free access to sterile water during this period but were fasted from solid chow. All urine was collected, and the total urine output was recorded. The urine samples were centrifuged at 3000 rpm for 10 min at 4 °C, and the supernatant was collected for the determination of total urinary protein concentration via the pyrogallol red colorimetric method using a commercial kit (Nanjing Jiancheng Bioengineering Institute, Nanjing, China) according to the manufacturer’s instructions. The absorbance was measured at 596 nm using a microplate reader (Bio-Rad, Hercules, CA, USA), and the urinary protein concentration was calculated based on a standard curve.

### 2.7. Tissue Collection

On GD20, all rats were anesthetized with 1% sodium pentobarbital (40 mg/kg, intraperitoneal injection) and sacrificed by cervical dislocation. The abdominal cavity was rapidly opened on an ice tray (4 °C), and decidual tissue, fetuses, placentas, livers, and kidneys were collected immediately. The entire uterus was removed, longitudinally incised along the uterine horn, and embryonic loss was observed and recorded. Fetal weight and body length were measured and recorded using an electronic balance and vernier caliper, respectively; placental weight and diameter were measured and recorded similarly. Three placentas and three fetuses were randomly selected from each dam for subsequent histological and immunofluorescence analysis.

Decidual tissues were carefully isolated from embryonic and implantation sites, cut into small blocks: one part was fixed in 4% paraformaldehyde for 24 h for IHC and histological staining, and the other part was snap-frozen in liquid nitrogen and stored at −80 °C for subsequent mRNA and protein detection. The predefined primary outcome of this study was the effect of the Ghrelin/GHSR-1a axis on PE-like phenotypes, including blood pressure, proteinuria and placental/fetal development in rats; the detection of mRNA and protein in decidual tissues was set as the secondary outcome, and the relevant experiments are in progress and will be published in subsequent studies. Livers, kidneys, and placentas were fixed in 4% paraformaldehyde for 24 h, followed by routine paraffin embedding and sectioning (3 μm for liver/kidney, 5 μm for placenta) for histological and immunofluorescence analysis.

### 2.8. Histological Analysis of Liver, Kidney, and Placental Tissues

Paraffin sections of liver (cross-section), kidney (cross-section), and placental implantation sites (perpendicular to the fetal axis) were subjected to hematoxylin–eosin (H&E) and periodic acid–Schiff (PAS) staining for histological analysis. For H&E staining, sections were dewaxed, rehydrated, stained with hematoxylin for 5 min, differentiated with 1% hydrochloric acid–ethanol, blued with ammonia water, stained with eosin for 1 min, dehydrated, cleared, and mounted. For PAS staining, sections were dewaxed, rehydrated, oxidized with 1% periodic acid for 10 min, rinsed with distilled water, stained with Schiff’s reagent for 20 min, counterstained with hematoxylin for 30 s, and then dehydrated, cleared, and mounted.

Sections containing maternal central arterial channels of the placenta were selected for analysis. Four to six random visual fields per section were captured under a light microscope (Olympus, Tokyo, Japan) at ×200 and ×400 magnifications. Histopathological changes were evaluated by two independent pathologists who were blinded to the group allocation, including hepatocyte morphology, hepatic sinusoid congestion, glomerular structure, renal tubular epithelial cell status, trophoblast morphology, and placental vascular basement membrane changes. The liver injury score (0–4 scale), glomerular damage score (0–5 scale), placental villous damage score (0–4 scale), and PAS-positive band continuity score (0–3 scale) used in this study were all validated classic scoring systems (the specific scoring criteria are shown in Table S1). Three consecutive sections per organ per animal were randomly selected for analysis, and four to six truly random visual fields per section were selected using the random point selection function of ImageJ software. The Kappa test was used to analyze the inter-rater reliability between the two pathologists, with a Kappa value > 0.8 indicating good consistency; disagreements were resolved by joint review of the sections and combination with the quantitative results of image analysis software.

### 2.9. Immunofluorescence Staining

Placental implantation site tissues were fixed in 4% paraformaldehyde at 4 °C for 4–6 h, then dehydrated in a graded sucrose solution (10%, 20%, 30%) and incubated in 30% sucrose at 4 °C overnight for cryoprotection. The tissues were embedded in optimal cutting temperature (OCT) compound (Sakura, Tokyo, Japan) and rapidly frozen in liquid nitrogen. Serial 4 μm thick cryosections were cut using a cryostat (Leica, Wetzlar, Germany) and stored at −80 °C until use.

For immunofluorescence staining, cryosections were air-dried at room temperature for 30 min, permeabilized with 0.3% Triton X-100 (Sigma-Aldrich, St. Louis, MO, USA) in PBS for 15 min, and blocked with blocking buffer (5% bovine serum albumin + 0.3% Triton X-100 in PBS) at room temperature for 45 min. The sections were incubated with primary antibodies against α-SMA (CY1132S, 1:200), α-CK7 (250022, 1:200), CD86 (R380350, 1:100), and CD163 (bsm-54015R, 1:100) at 4 °C overnight. After washing three times with PBS (5 min each), the sections were incubated with Alexa Fluor 488/Alexa Fluor 594-conjugated fluorescent secondary antibodies (1:500, Invitrogen, Carlsbad, CA, USA) at room temperature for 2 h in the dark. Nuclei were counterstained with 4′,6-diamidino-2-phenylindole (DAPI, 1:1000, Invitrogen) for 10 min. The sections were sealed with anti-fluorescence quenching mounting medium (Beyotime, Shanghai, China). PBS was used instead of primary antibodies as the negative control.

Fluorescent images were captured under a laser scanning confocal microscope (Zeiss, Oberkochen, Germany) at ×200 and ×400 magnifications with fixed laser intensity, exposure time and gain to ensure consistency across groups. Four to six random visual fields per section were selected for quantitative analysis using ImageJ software. The quantitative indicators included the mean fluorescence intensity (MFI) and the positive cell area fraction of the target proteins. The automatic segmentation function of ImageJ was used to separate the positive fluorescent regions from the background based on the fluorescence threshold, and the rolling ball method was used for background subtraction. All quantitative analyses were performed by two independent experimenters blinded to the group allocation, and the average value was taken as the final result.

### 2.10. Statistical Analysis

All experimental data were collated and analyzed using SPSS 18.0 statistical software (IBM, Armonk, NY, USA) and GraphPad Prism 9.0 (GraphPad Software, San Diego, CA, USA). All measurement data were first tested for normality (Shapiro–Wilk test) and homogeneity of variance (Levene test); data conforming to the normal distribution and homogeneity of variance were expressed as mean ± standard deviation (x ± s) for descriptive statistics and mean ± standard error of the mean (mean ± SEM) for statistical graphs and inferential statistics; non-conforming data were analyzed using non-parametric tests (Kruskal–Wallis H test). The experimental unit of the clinical study was the patient, and the experimental unit of the animal study was the dam; the detection results of placentas and fetuses from the same dam were averaged and then included in statistical analysis to avoid the pseudoreplication problem in litter-bearing species. Comparisons between two groups were performed using the independent samples *t*-test, and comparisons among multiple groups were performed using one-way analysis of variance (ANOVA) or repeated-measures ANOVA; the Mauchly test was used to verify the sphericity assumption of repeated-measures ANOVA, and the Greenhouse-Geisser correction was used when the sphericity assumption was not satisfied. The Bonferroni post hoc test was used for pairwise multiple comparisons to reduce the risk of false positives, and Bonferroni correction was applied for multiple outcome indicators to correct for multiple comparisons. Categorical data were expressed as n (%) and compared using the chi-square test or Fisher’s exact probability test. A two-tailed *p* value < 0.05 was considered statistically significant.

## 3. Results

### 3.1. Baseline Clinical Characteristics of the Study Population

There were no significant differences in maternal age, systolic/diastolic blood pressure, smoking history, or fetal birth weight between the mild PE group and the healthy control group (all *p* > 0.05, Table 1). Compared with the control group, patients with severe PE had a significantly higher baseline BMI and blood pressure, earlier gestational age at delivery, and a higher rate of preterm birth (all *p* < 0.05); these differences remained statistically significant after multivariate adjustment for maternal age and parity (all *p* < 0.05), which is consistent with clinical evidence that obesity is an independent risk factor for severe PE and preterm birth is a common complication of severe PE; no significant differences were observed in maternal age and fetal birth weight (all *p* > 0.05). Additionally, the severe PE group had a significantly earlier gestational age at delivery than the mild PE group (*p* < 0.01). All neonates had an Apgar score of 10 at 1 min, indicating no acute neonatal asphyxia in any group.

### 3.2. Ghrelin and GHSR-1a Are Upregulated in Decidual Tissues of PE Patients in a Severity-Dependent Manner

**Figure 1:**
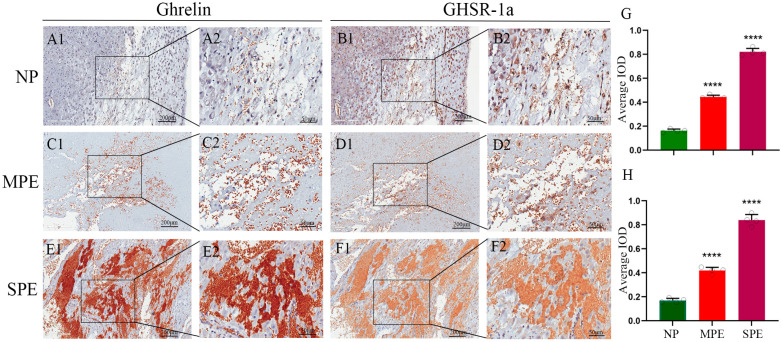
Expression Levels of Ghrelin and GHSR-1a in Human Decidual Tissues. (**A1**,**A2**) Ghrelin expression in the control group (Scale bar: 200 μm (**A1**); 50 μm (**A2**)); (**B1**,**B2**) GHSR-1a expression in the control group (Scale bar: 200 μm (**B1**); 50 μm (**B2**)); (**C1**,**C2**) Ghrelin expression in the mild PE group (Scale bar: 200 μm (**C1**); 50 μm (**C2**)); (**D1**,**D2**) GHSR-1a expression in the mild PE group (Scale bar: 200 μm (**D1**); 50 μm (**D2**)); (**E1**,**E2**) Ghrelin expression in the severe PE group (Scale bar: 200 μm (**E1**); 50 μm (**E2**)); (**F1**,**F2**) GHSR-1a expression in the severe PE group (Scale bar: 200 μm (**F1**); 50 μm (**F2**)). (**G**) Quantitative analysis of the average integrated optical density (IOD) of Ghrelin; (**H**) quantitative analysis of the average IOD of GHSR-1a. The white dots in the figure represent the individual raw data points of each sample. **** *p* < 0.0001 vs. Control.

IHC staining was performed to detect the expression and localization of Ghrelin and GHSR-1a in human decidual tissues (Figure 1). Ghrelin and GHSR-1a were mainly expressed in the cytoplasm and cell membrane of decidual stromal cells and villous trophoblasts at the maternal–fetal interface, with the strongest positive staining in the decidual layer. Univariate analysis revealed that the protein expression levels of Ghrelin and GHSR-1a were significantly higher in the PE group than in the control group (both *p* < 0.0001). Among PE patients, the severe PE group exhibited significantly higher expression of Ghrelin and GHSR-1a than the mild PE group (both *p* < 0.0001). This severity-dependent upregulation of the Ghrelin/GHSR-1a axis in PE decidual tissues was contrary to our initial hypothesis, suggesting a potential compensatory protective mechanism of the maternal body in response to PE-associated pathological stress at the maternal–fetal interface.

### 3.3. Ghrelin Mitigates Systemic Hypertension and Renal Impairment in PE Rats

**Figure 2:**
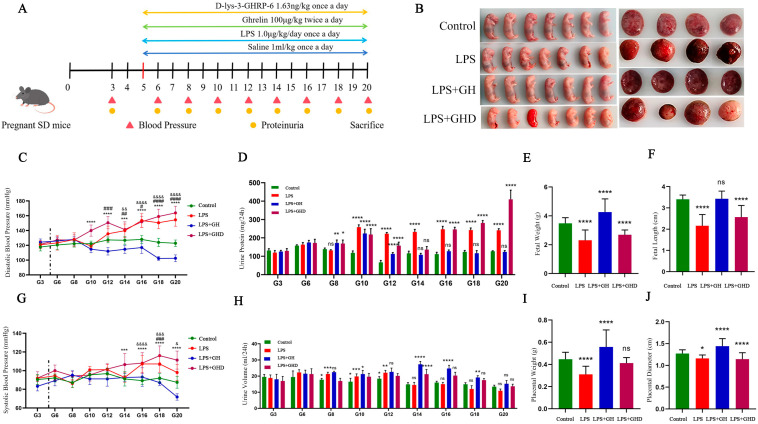
Ghrelin alleviates LPS-induced PE-like phenotypes in pregnant rats via the GHSR-1a axis. (**A**) Schematic of the animal experimental design and intervention protocol; (**B**) representative morphological images of placentas (**left**) and fetuses (**right**) in each group; (**C**,**G**) dynamic changes in systolic blood pressure (SBP) and diastolic blood pressure (DBP) from GD3 to GD20; (**D**,**H**) dynamic changes in urinary protein and urine output (14 h fasting collection) from GD3 to GD20; (**E**) quantitative analysis of fetal weight; (**F**) quantitative analysis of fetal length; (**I**) quantitative analysis of placental weight; (**J**) quantitative analysis of placental diameter. The vertical dash line indicates the time point of drug administration on G5. &: Control vs. LPS; #: Control vs. LPS+Ghrelin; *: Control vs. LPS+Ghrelin+D-lys-3-GHRP-6; data are expressed as mean ± SEM; &, *p* < 0.05; &&, *p* < 0.01; &&&, *p* < 0.001; &&&&, *p* < 0.0001; #, *p* < 0.05; ##, *p* < 0.01; ###, *p* < 0.001; ####, *p* < 0.0001; * *p* < 0.05, ** *p* < 0.01, *** *p* < 0.001, **** *p* < 0.0001; ns, no significant. GD: Gestational Day; LPS: Lipopolysaccharide; GHD: D-lys-3-GHRP-6. Scale bar in (**B**): 1 cm.

To validate the functional role of the Ghrelin/GHSR-1a axis in PE pathogenesis and confirm its protective effect, we established an LPS-induced PE-like rat model (Figure 2A) and further investigated the regulatory effects of exogenous Ghrelin supplementation and GHSR-1a blockade on PE-related phenotypes. Compared with control pregnant rats receiving saline, tail vein injection of LPS resulted in significantly elevated SBP and DBP, increased proteinuria, and impaired fetal–placental development. As shown in Figure 2C,G, DBP and SBP remained stable throughout pregnancy in the control group. In contrast, LPS-treated rats exhibited a significant increase in DBP starting at G12, which persisted until term, and fluctuating elevations in SBP. Co-administration of Ghrelin with LPS reversed the LPS-induced hypertension, reducing both DBP and SBP to control levels. Co-treatment with D-Lys3-GHRP-6 abrogated the antihypertensive effect of Ghrelin, resulting in blood pressure levels comparable to those in the LPS group. We next evaluated renal function by measuring urine output and proteinuria. Compared with controls, LPS-treated rats showed reduced urine output starting at G8 (Figure 2H) and significantly increased proteinuria starting at G10 (Figure 2D), indicating renal dysfunction. Ghrelin treatment ameliorated these renal impairments, increasing urine output and reducing proteinuria. Co-administration of D-Lys3-GHRP-6 blocked the renoprotective effect of Ghrelin, with proteinuria levels similar to those in the LPS group and no significant change in urine output. Assessment of fetal and placental development at G20 revealed that LPS treatment caused fetal growth restriction, characterized by limb malformations (Figure 2B), reduced fetal weight (Figure 2E), and smaller fetal length (Figure 2F). These adverse fetal outcomes were prevented by Ghrelin treatment, with fetal morphology, weight, and length comparable to those in the control group. Co-treatment with D-Lys3-GHRP-6 abolished the fetal-protective effect of Ghrelin, resulting in fetal abnormalities similar to those in the LPS group. For placental development, LPS treatment led to irregular placental morphology, pale color, reduced weight (Figure 2I), and smaller diameter (Figure 2J), indicative of placental ischemia. Ghrelin treatment restored normal placental morphology, weight, and diameter, while co-administration of D-Lys3-GHRP-6 reversed these beneficial effects, resulting in placental defects similar to those in the LPS group (*p* < 0.05).

### 3.4. Ghrelin Attenuates LPS-Induced Maternal Liver, Kidney, and Placental Pathological Damage

**Figure 3:**
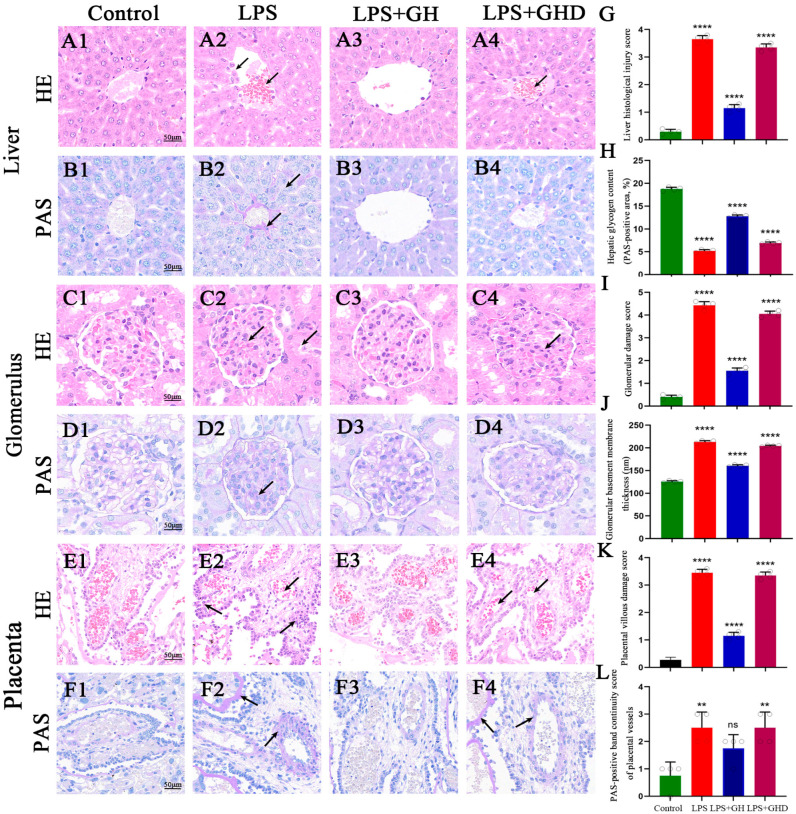
Ghrelin attenuates LPS-induced liver, kidney, and placental pathological damage in PE-like rats. (**A1**–**F1**) H&E and PAS staining of liver, kidney, and placenta in the control group (scale bar: 50 μm); (**A2**,**B2**) LPS-induced liver damage: hepatocyte swelling/detachment, hepatic sinusoid congestion (H&E, black arrows), and reduced glycogen and fibrin deposition (PAS, black arrows); (**C2**,**D2**) LPS-induced kidney damage: glomerular swelling, renal tubular epithelial cell necrosis (H&E, black arrows), and glomerular subendothelial fibrinoid deposition (PAS, black arrows); (**E2**,**F2**) LPS-induced placental damage: trophoblast hyperplasia/detachment, reduced intravascular erythrocytes (H&E, black arrows), thickened basement membrane, and vascular fibrinoid deposition (PAS, black arrows); (**A3**–**F3**) Ghrelin treatment restores normal histological structures of liver, kidney, and placenta; (**A4**–**F4**) D-lys-3-GHRP-6 abrogates Ghrelin’s protective effect, with pathological damage similar to the LPS group; (**G**) liver histological injury score (0–4 scale); (**H**) relative hepatic glycogen content (PAS-positive area, %); (**I**) glomerular damage score (0–5 scale); (**J**) glomerular basement membrane thickness (nm); (**K**) placental villous damage score (0–4 scale); (**L**) PAS-positive band continuity score of placental vessels (0–3 scale). The white dots in the figure represent the individual raw data points of each sample. ** *p* < 0.01, **** *p* < 0.0001 vs. Control; ns, no significant; H&E: Hematoxylin–Eosin; PAS: Periodic Acid–Schiff; scale bar: 50 μm for all images.

Given that PE is clinically characterized by systemic multi-organ damage and placental dysfunction as the core pathological change, we next evaluated the histological damage of maternal liver, kidney and placenta in each group by means of H&E and PAS staining (Figure 3). The control group showed normal histological structures of the liver, kidney, and placenta: the liver had intact hepatocyte cords and no hepatic sinusoid congestion; the kidney had normal glomerular and renal tubular structures with no inflammatory cell infiltration; the placenta had regular trophoblast layers, intact vascular structures, and no abnormal deposition.

In the LPS group, severe histological damage was observed in all three tissues: (1) Liver: hepatocyte swelling and detachment, hepatic sinusoid congestion with red blood cell accumulation, and reduced glycogen content in hepatic sinusoidal endothelial cells accompanied by fibrin deposition (Figure 3(A2,B2),G,H); (2) Kidney: glomerular swelling and atrophy, renal tubular epithelial cell necrosis and disintegration, and fibrinoid deposition in the glomerular endothelial subendothelium (Figure 3(C2,D2),I,J); (3) Placenta: trophoblast hyperplasia and detachment, reduced intravascular erythrocytes, thickened vascular basement membranes with discontinuous PAS-positive bands, and focal fibrinoid deposition in vessel walls (Figure 3(E2,F2),K,L).

Ghrelin co-treatment effectively reversed all of these LPS-induced pathological changes, with the histological structures of the liver, kidney, and placenta restored to near-normal levels (Figure 3(A3–F3)), consistent with the control group. However, co-administration of D-lys-3-GHRP-6 completely abolished the protective effect of Ghrelin, and the LPS+Ghrelin+D-lys-3-GHRP-6 group exhibited histological lesions similar to the LPS group (Figure 3(A4–F4)). These results confirm that Ghrelin mitigates LPS-induced maternal multi-organ and placental damage in a GHSR-1a-dependent manner.

### 3.5. Ghrelin Restores LPS-Impaired Placental Vascularization by Upregulating Laminin Expression

**Figure 4:**
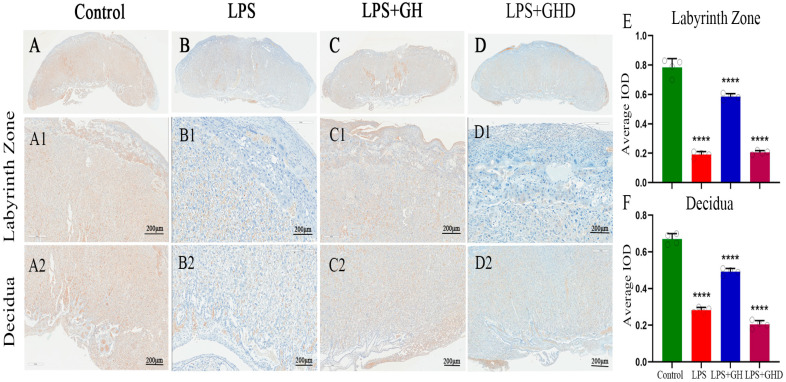
Ghrelin upregulates laminin expression to restore LPS-impaired placental vascularization. (**A**) Laminin expression in the control group; (**A1**,**A2**) laminin expression in the labyrinth zone and decidual layer in the control group; (**B**) laminin expression in the LPS group; (**B1**,**B2**) laminin expression in the labyrinth zone and decidual layer in the LPS group; (**C**) laminin expression in the LPS+Ghrelin group; (**C1**,**C2**) laminin expression in the labyrinth zone and decidual layer in the LPS+Ghrelin group; (**D**) laminin expression in the LPS+Ghrelin+D-lys-3-GHRP-6 group; (**D1**,**D2**) laminin expression in the labyrinth zone and decidual layer in the LPS+Ghrelin+D-lys-3-GHRP-6 group; (**E**) quantitative analysis of the average integrated optical density (IOD) of laminin in the labyrinth zone; (**F**) quantitative analysis of the average IOD of laminin in decidual layer. **** *p* < 0.0001 vs. Control; scale bar: 200 μm.

Placental vascular dysfunction is the key link of PE-induced placental hypoperfusion, and laminin is a core component of the vascular basement membrane regulating placental vascularization. We therefore detected laminin expression in placental tissues to explore the effect of the Ghrelin/GHSR-1a axis on placental vascular integrity (Figure 4). In the control group, laminin was highly expressed in the decidual layer and placental labyrinth layer, with continuous and uniform staining along the vascular basement membrane (Figure 4A,(A1,A2)), indicating intact placental vascularization. In the LPS group, laminin expression was significantly downregulated in both the decidual layer and labyrinth layer, with discontinuous and weak staining (Figure 4B,(B1,B2), *p* < 0.0001), suggesting impaired placental vascular network formation and defective capillary development. Ghrelin co-treatment restored laminin expression to control levels in the placental decidual and labyrinth layers (Figure 4C,(C1,C2), *p* < 0.0001), indicating improved placental vascularization. Co-administration of D-lys-3-GHRP-6 blocked the upregulatory effect of Ghrelin on laminin, and the laminin expression in the LPS+Ghrelin+D-lys-3-GHRP-6 group was similar to that in the LPS group (Figure 4D,(D1,D2), *p* < 0.0001). These results suggest that Ghrelin promotes placental vascularization and improves placental function by upregulating laminin expression via the GHSR-1a axis.

### 3.6. Ghrelin Reverses LPS-Induced Abnormal Placental Tissue Remodeling by Regulating α-CK7 and α-SMA Expression

**Figure 5:**
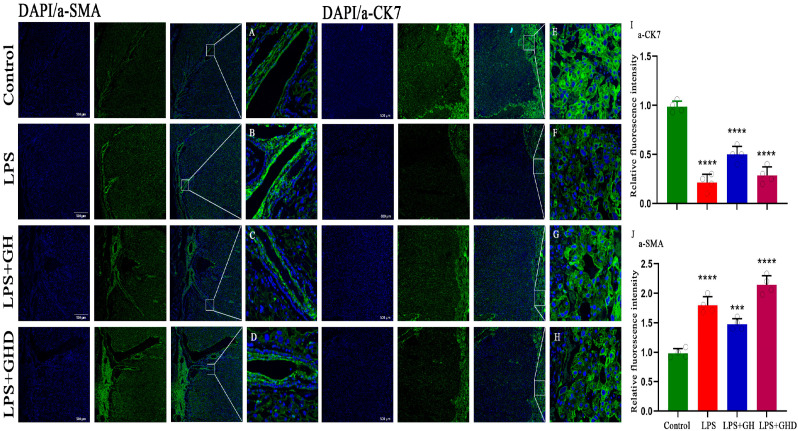
Ghrelin regulates α-CK7 and α-SMA expression to reverse LPS-induced abnormal placental tissue remodeling. (**A**–**D**) Representative immunofluorescence images of α-SMA (green) in placental tissues of the Control, LPS, LPS+Ghrelin, and LPS+Ghrelin+D-lys-3-GHRP-6 groups (nuclei stained with DAPI, blue; scale bar: 500 μm); (**E**–**H**) representative immunofluorescence images of α-CK7 (green) in placental tissues of each group (nuclei stained with DAPI, blue; scale bar: 500 μm); (**I**) quantitative analysis of α-CK7 relative fluorescence intensity; (**J**) quantitative analysis of α-SMA relative fluorescence intensity. Data are expressed as mean ± SEM; *** *p* < 0.001, **** *p* < 0.0001 vs. Control; α-CK7: α-cytokeratin 7; α-SMA: α-smooth muscle actin; DAPI: 4′,6-diamidino-2-phenylindole.

Based on the improvement of placental vascularization by Ghrelin, we further detected the expression of trophoblast marker α-CK7 and spiral artery remodeling marker α-SMA to clarify the regulatory effect of the axis on placental tissue remodeling (Figure 5). Compared with the control group, the LPS group exhibited a significant downregulation of α-CK7 expression (Figure 5F,I, *p* < 0.0001) and a significant upregulation of α-SMA expression (Figure 5B,J, *p* < 0.0001), indicating impaired trophoblast integrity and abnormal activation of myofibroblasts, which are associated with defective trophoblast invasion and spiral artery remodeling.

Ghrelin co-treatment significantly reversed these abnormal expressions: α-CK7 expression was upregulated to control levels (*p* < 0.0001), and α-SMA expression was downregulated to near-normal levels (*p* < 0.001). Notably, D-lys-3-GHRP-6 co-administration abolished the effects of Ghrelin, with α-CK7 and α-SMA expression returning to levels comparable to the LPS group (both *p* < 0.0001). These results confirm that Ghrelin alleviates LPS-induced placental tissue remodeling disorder and trophoblast damage by regulating α-CK7 and α-SMA expression via the GHSR-1a axis, thereby promoting trophoblast invasion and spiral artery remodeling.

### 3.7. Ghrelin Modulates Decidual Macrophage Infiltration and Marker Expression in LPS-Induced PE-like Rats

**Figure 6:**
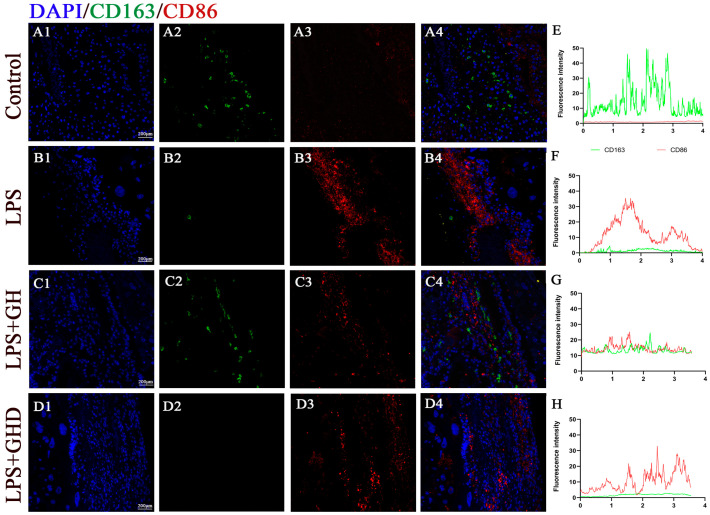
Ghrelin modulates decidual macrophage infiltration and phenotypic marker expression in LPS-induced PE-like rats. (**A1**–**D4**) Representative immunofluorescence co-staining images of CD163 (green, M2 marker) and CD86 (red, M1 marker) in placental decidual tissues of each group (nuclei stained with DAPI, blue; scale bar: 200 μm); (**A1**–**D1**) merged images of CD163, CD86, and DAPI; (**A2**–**D2**) CD163 single-channel images; (**A3**–**D3**) CD86 single-channel images; (**A4**–**D4**) DAPI single-channel images; (**E**–**H**) corresponding fluorescence intensity profiles of CD163 (green) and CD86 (red) across placental tissue sections, reflecting the distribution and relative abundance of pro-inflammatory and anti-inflammatory macrophage phenotypes in each group.

Decidual macrophage phenotypic imbalance is the core immune mechanism of PE-induced placental dysfunction and spiral artery remodeling defects. We then investigated the effect of the Ghrelin/GHSR-1a axis on decidual macrophage infiltration and marker expression, the key upstream immune regulator of placental function. We performed immunofluorescence co-staining of CD86 (M1 marker) and CD163 (M2 marker) to evaluate macrophage-associated phenotypic changes (Figure 6). In the control group, CD163-positive M2 macrophages were the predominant subtype at the maternal–fetal interface, with minimal CD86-positive M1 macrophage infiltration, indicating a normal M1/M2 polarization balance (Figure 6(A1–A4)). In the LPS group, a marked shift toward M1 polarization was observed, characterized by significantly increased CD86 fluorescence intensity and decreased CD163 fluorescence intensity (Figure 6(B1–B4)), suggesting enhanced pro-inflammatory macrophage infiltration and an imbalanced immune microenvironment.

Ghrelin co-treatment significantly attenuated LPS-induced M1 polarization: CD86 expression was significantly downregulated, and CD163 expression was restored to control levels (Figure 6(C1–C4)), reducing pro-inflammatory macrophage infiltration and favoring an anti-inflammatory immune profile. The LPS+Ghrelin+D-lys-3-GHRP-6 group exhibited a similar polarization pattern to the LPS group, with significantly increased CD86 expression and significantly decreased CD163 expression, which indicated that the GHSR-1a antagonist completely abrogated the regulatory effect of Ghrelin on decidual macrophage-related marker expression. The fluorescence intensity profiles further confirmed these results (Figure 6E–H): the LPS group had a high CD86 intensity peak and low CD163 intensity, while the LPS+Ghrelin groups had CD86/CD163 intensity profiles comparable to the control group. These results suggest that Ghrelin modulates decidual macrophage infiltration and phenotypic marker expression in a GHSR-1a-dependent manner, thereby potentially improving the immune microenvironment at the maternal–fetal interface in PE-like rats.

### 3.8. Ghrelin Regulates the Expression of the Ghrelin/GHSR-1a Axis in Decidual Tissues of LPS-Induced PE-like Rats

**Figure 7:**
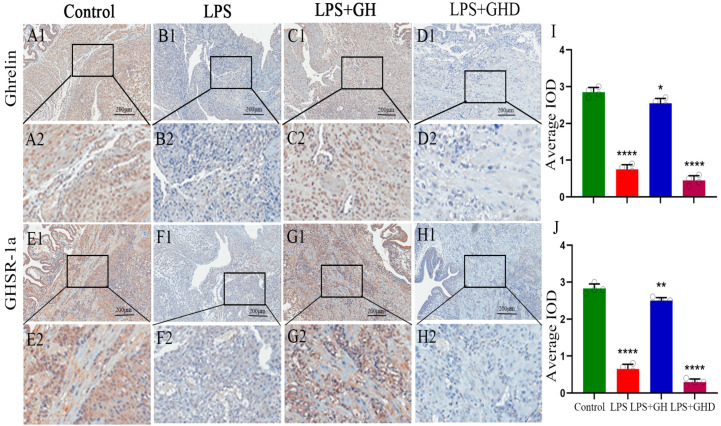
Expression of the Ghrelin/GHSR-1a axis in decidual tissues of LPS-induced PE-like rats. (**A1**–**D2**) Representative IHC images of Ghrelin expression in the Control, LPS, LPS+Ghrelin, and LPS+Ghrelin+D-lys-3-GHRP-6 groups (scale bar: 200 μm); (**E1**–**H2**) representative IHC images of GHSR-1a expression in each group (scale bar: 200 μm); (**I**) quantitative analysis of Ghrelin relative expression; (**J**) quantitative analysis of GHSR-1a relative expression. Brown–yellow staining indicates positive expression; data are expressed as mean ± SEM; * *p* < 0.05, ** *p* < 0.01, **** *p* < 0.0001 vs. Control,.

To further confirm the regulatory relationship between the Ghrelin/GHSR-1a axis and the above protective effects, we detected the expression of Ghrelin and GHSR-1a in decidual tissues of PE-like rats and verified the axis’s self-regulation pattern (Figure 7). Consistent with the clinical results, the LPS group exhibited significantly lower expression of Ghrelin and GHSR-1a than the control group (both **** *p* < 0.0001), indicating a compensatory downregulation of the Ghrelin/GHSR-1a axis in the rat PE-like model. Exogenous Ghrelin supplementation further upregulated the expression of Ghrelin (*p* < 0.05) and GHSR-1a (*p* < 0.01) in decidual tissues, suggesting a positive feedback regulation of the axis. Co-administration of D-lys-3-GHRP-6 completely blocked the upregulatory effect of exogenous Ghrelin, and the expression of Ghrelin and GHSR-1a was significantly downregulated to levels comparable to the LPS group (both *p* < 0.0001). These results suggest that the Ghrelin/GHSR-1a axis is compensatorily regulated in both human PE and rat PE-like models, and exogenous Ghrelin modulates the expression of this axis in decidual tissues in a GHSR-1a-dependent manner.

## 4. Discussion

Preeclampsia (PE), a leading cause of maternal and perinatal morbidity and mortality, is characterized by immune dysregulation at the maternal–fetal interface and placental dysfunction [14]. Decidual macrophages (DMs), as key immune cells in this microenvironment, maintain pregnancy homeostasis through M1/M2-related phenotypic balance. Disruption of this balance—increased pro-inflammatory macrophage dominance—contributes to PE pathogenesis by inhibiting trophoblast invasion and spiral artery (SA) remodeling [22,23,24]. Ghrelin, a multifunctional peptide, exerts protective effects in various inflammatory diseases via its receptor GHSR-1a, but its role in regulating DM phenotypic profiles in PE remains unclear. This study explores the expression of the Ghrelin/GHSR-1a axis in PE and its regulatory effect on DM infiltration and phenotypic marker expression, providing new preliminary insights into PE’s mechanisms.

Our clinical data revealed a compensatory increase in Ghrelin and GHSR-1a expression in decidual tissues with escalating PE severity: severe PE patients had significantly higher expression than mild PE patients, who in turn had higher levels than healthy pregnant women. This contrasts with our previous hypothesis of downregulated Ghrelin expression in PE but aligns with reports of elevated Ghrelin in late-onset PE, suggesting a compensatory response to placental dysfunction [25]. Notably, PE patients had lower gestational age at delivery and higher BMI than healthy controls, consistent with clinical observations that obesity is a PE risk factor and preterm delivery is a common complication. The compensatory upregulation of Ghrelin/GHSR-1a may reflect the body’s attempt to mitigate PE-related damage, laying the foundation for subsequent animal experiments.

Using an LPS-induced PE rat model, we confirmed that Ghrelin intervention alleviates PE-related symptoms in a GHSR-1a-dependent manner. LPS-induced rats exhibited hypertension, proteinuria, impaired placental development, and fetal growth restriction—classic PE phenotypes [26,27]. Ghrelin supplementation significantly reduced blood pressure and urinary protein levels, restored placental weight and structure, and improved fetal weight and length. However, co-administration of D-Lys3-GHRP-6, a specific GHSR-1a antagonist, abrogated these protective effects, confirming that Ghrelin’s actions are mediated by GHSR-1a. This is consistent with previous studies showing Ghrelin regulates vascular function and inflammation via GHSR-1a [28,29,30], supporting the potential value of this axis for further mechanistic investigation in PE.

Notably, a striking divergence in Ghrelin expression was observed between human and rat decidual tissues: while Ghrelin was upregulated in human PE decidual tissues, it was significantly downregulated in the LPS-induced rat PE-like model compared to normal pregnant controls. This discrepancy is not unexpected and likely arises from fundamental differences in species-specific pathophysiology, the timing of pathological insults, and the inherent limitations of the animal model [31]. This paradoxical divergence likely reflects the distinct kinetic profiles of human chronic PE versus the acute systemic inflammatory onslaught in the LPS model, where the latter’s severity may overwhelm the initial compensatory capacity of the Ghrelin system. Human PE is a chronic, multifactorial syndrome driven by sustained placental ischemia and low-grade inflammation over weeks to months [32], allowing sufficient time for the maternal–fetal interface to mount adaptive compensatory responses [33]—with Ghrelin upregulation serving as a cell-autonomous protective mechanism in decidual stromal cells and trophoblasts. In contrast, the LPS-induced rat model mimics only the acute, systemic pro-inflammatory features of human PE, with a rapid and severe inflammatory challenge initiated early in gestation that overwhelms endogenous adaptive pathways, leading to a failure of compensatory Ghrelin upregulation and subsequent downregulation of the axis [34]. The results of this animal study need to be verified by large-sample, multi-center clinical studies before clinical translation.

PE often involves multi-organ damage, and our study demonstrated that LPS-induced PE rats had significant pathological changes in the liver, kidneys, and placenta, including hepatocellular edema, glomerular mesangial hyperplasia, and placental villous edema. Ghrelin intervention reversed these damages, with liver, kidney, and placental structures approaching those of the control group, while D-Lys3-GHRP-6 blocked this protective effect. These findings suggest the Ghrelin/GHSR-1a axis may mitigate PE-induced multi-organ injury, possibly by inhibiting inflammatory responses and oxidative stress. The placenta, as the core pathological site of PE [35], showed restored structure and function after Ghrelin treatment, which may improve maternal–fetal nutrient exchange and fetal development.

Zhang L et al. found that endogenous ghrelin promotes acute inflammatory responses in LPS-induced ARDS by regulating macrophage activity via GHS-R1a signaling, identifying macrophage GHS-R1a as a potential therapeutic target for this refractory disease with dysregulated pulmonary inflammation and no effective pharmacological treatments [36]. Immunofluorescence results indicated that LPS-induced PE rats had increased M1 macrophage markers (CD86) and decreased M2 markers (CD163) in decidual tissues, which is consistent with altered macrophage infiltration and a shift toward pro-inflammatory phenotypes. Ghrelin intervention reduced CD86 expression and increased CD163 expression, alleviating pro-inflammatory macrophage dominance, while D-Lys3-GHRP-6 blocked this effect. This suggests that Ghrelin regulates decidual macrophage infiltration and phenotypic marker expression via GHSR-1a.

Macrophage phenotypic profiles are closely related to trophoblast invasion and spiral artery (SA) remodeling: anti-inflammatory macrophage phenotypes promote these processes, while pro-inflammatory phenotypes inhibit them. Thus, Ghrelin may improve placental perfusion and function by reducing pro-inflammatory macrophage infiltration and favoring an anti-inflammatory decidual microenvironment, thereby potentially alleviating PE progression. Additionally, Ghrelin upregulated α-SMA and CK7 expression in placental tissues, suggesting enhanced SA remodeling and trophoblast invasion—key mechanisms underlying its protective effects.

This study has several limitations that need to be acknowledged. First, the clinical sample size is relatively small (n = 22), and although post-hoc power analysis confirmed sufficient statistical power for key indicators, the results still need to be validated by large-sample, multi-center prospective studies. Second, the study was restricted to cesarean section patients, which may introduce selection bias and limit the generalizability of the results to vaginal delivery populations; future studies should include vaginal delivery cases to eliminate the confounding effect of delivery mode. Third, the study only evaluated CD86 and CD163 without pan-macrophage markers such as CD68 or Iba1, which prevents definitive distinction between macrophage polarization and changes in total macrophage infiltration; interpretations should therefore be made with caution. Fourth, the LPS-induced PE-like rat model only mimics the acute inflammatory characteristics of human PE and cannot fully replicate the chronic placental ischemia and hypoxia process of human PE; subsequent studies can use other PE models, such as the reduced uterine perfusion pressure model, for cross-validation. Finally, the long-term effects of exogenous Ghrelin supplementation on maternal and offspring metabolic and cardiovascular functions remain unclear and require further long-term follow-up studies.

## 5. Conclusions

This study demonstrates that the Ghrelin/GHSR-1a axis is compensatorily upregulated in decidual tissues of PE patients. In the LPS-induced PE rat model, Ghrelin acts via GHSR-1a to alleviate hypertension, proteinuria, and multi-organ pathological damage and improve placental and fetal development. Meanwhile, Ghrelin regulates decidual macrophage infiltration and phenotypic marker expression to modulate the immune profile at the maternal–fetal interface. Administration of D-Lys3-GHRP-6 abolishes the protective effects of Ghrelin, confirming the dependence of these effects on the Ghrelin/GHSR-1a axis.

## Figures and Tables

**Table 1:** Baseline Clinical Characteristics and Perinatal Outcomes of Healthy Pregnant Women and Preeclampsia (PE) Patients.

Variable	Healthy Pregnancy (n = 10)	Mild PE (n = 7)	Severe PE (n = 5)	PE (n = 12)	* *p* Value	** *p* Value	*** *p* Value	^◎^* p* Value
Maternal age (year)	30.7 ± 4.3	28.7 ± 4.8	29.6 ± 5.7	29.1 ± 5.2	0.42	0.70	0.79	0.46
BMI at test (kg/m^2^)	21.2 ± 2.9	23.9 ± 3.5	27.3 ± 4.3	25.3 ± 4.2	0.13	0.01	0.19	0.02
Nulliparous (n, %)	4 (40.0%)	4 (57.1%)	5 (100.0%)	9 (75.0%)	0.52	0.02	0.11	0.11
Multiparous (n, %)	6 (60.0%)	3 (42.9%)	0 (0.0%)	3 (25.0%)	0.52	0.02	0.11	0.11
Smoker (n, %)	0	0	0	0	1.00	1.00	1.00	1.00
SBP (mmHg)	117.5 ± 5.8	142.3 ± 7.1	165.8 ± 9.4	151.2 ± 12.6	<0.001	0.002	<0.001	<0.001
DBP (mmHg)	71.2 ± 4.3	85.7 ± 5.2	102.4 ± 6.5	91.8 ± 9.7	<0.001	0.003	<0.001	<0.001
Gestational week at diagnosis (week)	N/A	37.2 ± 1.3	33.5 ± 4.1	35.7 ± 3.4	N/A	N/A	0.07	N/A
Gestational age at delivery (week)	39.2 ± 1.0	37.8 ± 1.0	35.9 ± 2.1	37.0 ± 1.8	0.02	0.002	0.08	0.004
Preterm birth (<37 weeks) (n, %)	0	0	2 (40.0%)	2 (16.7%)	1.00	0.03	0.08	0.19
Birth weight (g)	3383 ± 296	3224 ± 554	2496 ± 660	2920 ± 695	0.48	0.005	0.09	0.08
Cesarean section (n, %)	3 (30.0%)	5 (71.4%)	2 (40.0%)	7 (58.3%)	0.10	0.30	0.70	0.09

* *p* value: Mild PE vs. Control; ** *p* value: Severe PE vs. Control; *** *p* value: Severe PE vs. Mild PE; ^◎^ *p* value: Total PE vs. Control; BMI: Body Mass Index; SBP: Systolic Blood Pressure; DBP: Diastolic Blood Pressure; N/A: Not applicable.

## Data Availability

All data generated or analyzed during this study are available from the corresponding authors upon reasonable request.

## Supplementary Materials

The following supporting information can be downloaded at: https://www.mdpi.com/article/10.3390/biom16060809/s1, Figure S1: Participant screening and enrollment flowchart; Table S1: Specific Scoring Criteria for Histopathological Damage Assessment.

## Abbreviations

The following abbreviations are used in this manuscript:

PE	Preeclampsia
GHSR-1a	Growth hormone secretagogue receptor-1a
IHC	Immunohistochemistry
LPS	Lipopolysaccharide
FGR	Fetal growth restriction
SA	Spiral artery
DMs	Decidual macrophages
BMI	Body mass index
SBP	Systolic blood pressure
DBP	Diastolic blood pressure
PBS	Phosphate-buffered saline
HRP	Horseradish peroxidase
DAB	Diaminobenzidine
SD	Sprague Dawley
GD	Gestational day
SPF	Specific pathogen-free
H&E	Hematoxylin–eosin
PAS	Periodic acid–Schiff
OCT	Optimal cutting temperature
α-CK7	α-cytokeratin 7
α-SMA	α-smooth muscle actin
