# Supplementary material for: The Ghrelin/GHSR-1a Axis Attenuates Preeclampsia-like Features with Decidual Macrophage Reprogramming and Improved Placental Remodeling
Source: Biomolecules. 2026 May 29;16(6):809. doi: 10.3390/biom16060809 (PMC13296771; doi:10.3390/biom16060809)
Supplement: Supplementary file 1 [file biomolecules-16-00809-s001.zip › Table S1.pdf]

Table S1. Specific Scoring Criteria for Histopathological Damage Assessment

Histopathological Index	Scoring Scale	Score	Specific Criteria
**Liver Injury**	0–4 scale	0	No pathological damage; intact hepatocyte cords, no hepatic sinusoid congestion, normal glycogen content in hepatocytes
		1	Mild damage; slight swelling of individual hepatocytes, mild focal hepatic sinusoid congestion without red blood cell accumulation
		2	Moderate damage; diffuse mild hepatocyte swelling, moderate hepatic sinusoid congestion with local red blood cell accumulation, slight reduction of hepatic glycogen
		3	Severe damage; obvious hepatocyte swelling and partial detachment, severe hepatic sinusoid congestion with extensive red blood cell accumulation, significant reduction of hepatic glycogen, focal fibrin deposition
		4	Severe irreversible damage; massive hepatocyte necrosis and detachment, complete hepatic sinusoid congestion, almost no hepatic glycogen, extensive fibrin deposition in hepatic lobules
**Glomerular Damage**	0–5 scale	0	No pathological damage; normal glomerular structure, intact capillary loops, no mesangial hyperplasia, normal renal tubular epithelial cells
		1	Mild damage; slight mesangial cell proliferation (<25% of glomerular area), no thickening of glomerular basement membrane (GBM)
		2	Moderate mild damage; moderate mesangial hyperplasia (25%–50% of glomerular area), slight GBM thickening, no renal tubular epithelial cell damage
		3	Moderate severe damage; severe mesangial hyperplasia (>50% of glomerular area), obvious GBM thickening, mild swelling of renal tubular epithelial cells
		4	Severe damage; glomerular swelling and partial atrophy, focal fibrinoid deposition in glomerular

Histopathological Index	Scoring Scale	Score	Specific Criteria
			subendothelium, necrosis of individual renal tubular epithelial cells
		5	Severe irreversible damage; glomerular sclerosis and atrophy, extensive fibrinoid deposition in glomeruli, massive renal tubular epithelial cell necrosis and disintegration
**Placental Villous Damage**	0–4 scale	0	No pathological damage; regular placental villous structure, intact trophoblast layers, normal number of intravascular erythrocytes, no vascular basement membrane thickening
		1	Mild damage; slight irregularity of individual villi, intact trophoblast layers, slight reduction of intravascular erythrocytes
		2	Moderate damage; diffuse mild villous irregularity, partial trophoblast cell swelling, moderate reduction of intravascular erythrocytes, slight thickening of vascular basement membrane
		3	Severe damage; obvious villous hyperplasia and deformation, trophoblast cell detachment, severe reduction of intravascular erythrocytes, obvious thickening of vascular basement membrane, focal fibrinoid deposition in vessel walls
		4	Severe irreversible damage; massive villous atrophy and deformation, complete trophoblast layer detachment, almost no intravascular erythrocytes, severe thickening of vascular basement membrane, extensive fibrinoid deposition in vessel walls
**PAS-positive Band Continuity of Placental Vessels**	0–3 scale	0	Complete continuity; PAS-positive band of vascular basement membrane is continuous, uniform and intact without any interruption
		1	Mild discontinuity; focal slight interruption of PAS-positive band (<25% of vascular circumference), basically uniform thickness
		2	Moderate discontinuity; extensive interruption of PAS-positive band (25%–50% of vascular circumference), uneven thickness of the remaining band

**Histopathological Index**	**Scoring Scale**	**Score**	**Specific Criteria**
		3	Severe discontinuity; almost complete interruption of PAS-positive band (>50% of vascular circumference), obvious thinning and fragmentation of the remaining band

Table S1. Validated classic scoring systems for liver injury, glomerular damage, placental villous damage, and PAS-positive band continuity of placental vessels used in the study.
