# Supplementary figures and images for: The Ghrelin/GHSR-1a Axis Attenuates Preeclampsia-like Features with Decidual Macrophage Reprogramming and Improved Placental Remodeling
Source: Biomolecules. 2026 May 29;16(6):809. doi: 10.3390/biom16060809 (PMC13296771; doi:10.3390/biom16060809)

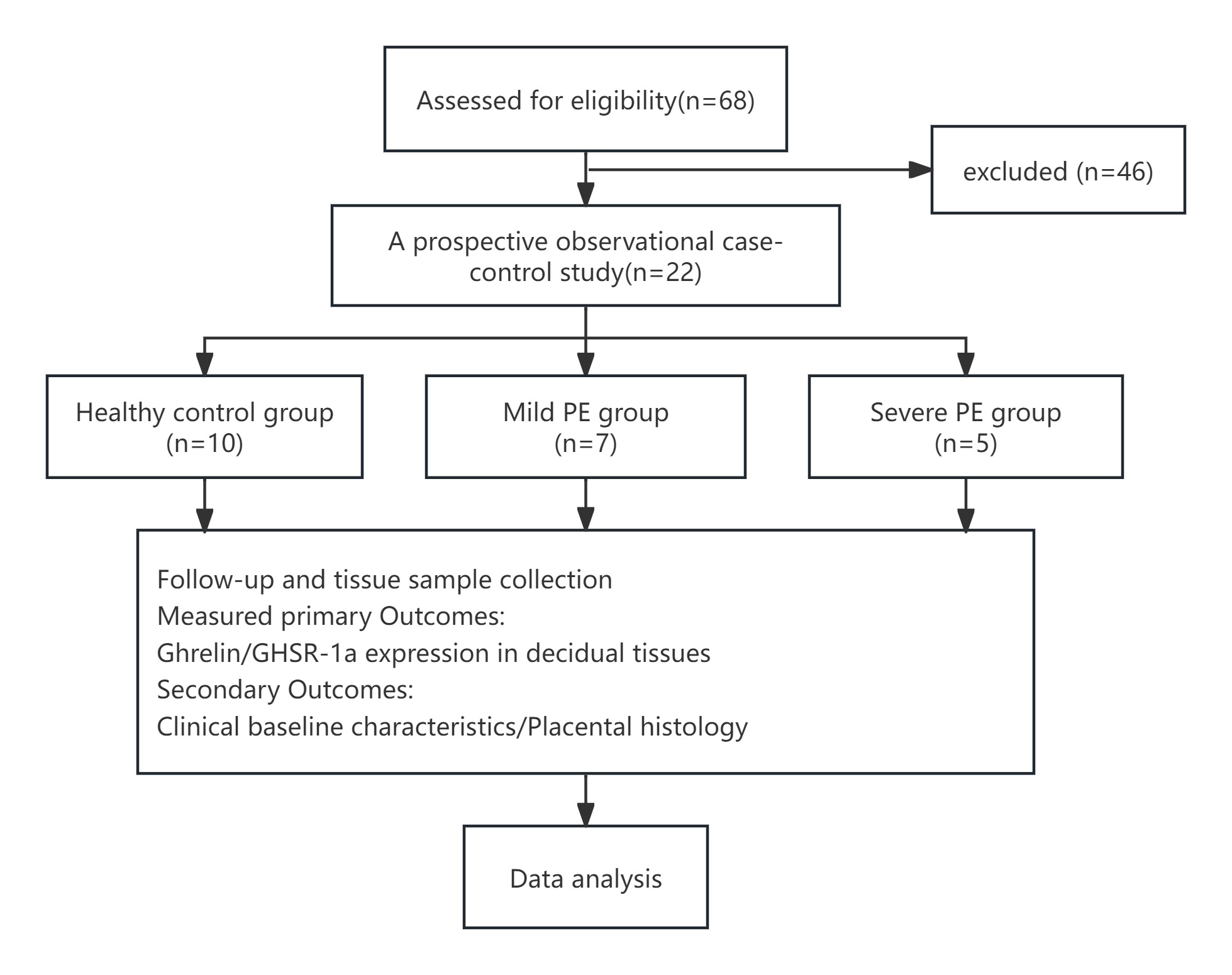

Supplement: Supplementary file 1 [file biomolecules-16-00809-s001.zip › Figure S1.jpg]
